# Hydroxychloroquine in the treatment of outpatients with mildly symptomatic COVID-19: a multi-center observational study

**DOI:** 10.1186/s12879-021-05773-w

**Published:** 2021-01-14

**Authors:** Andrew Ip, Jaeil Ahn, Yizhao Zhou, Andre H. Goy, Eric Hansen, Andrew L. Pecora, Brittany A. Sinclaire, Urszula Bednarz, Michael Marafelias, Ihor S. Sawczuk, Joseph P. Underwood, David M. Walker, Rajiv Prasad, Robert L. Sweeney, Marie G. Ponce, Samuel La Capra, Frank J. Cunningham, Arthur G. Calise, Bradley L. Pulver, Dominic Ruocco, Greggory E. Mojares, Michael P. Eagan, Kristy L. Ziontz, Paul Mastrokyriakos, Stuart L. Goldberg

**Affiliations:** 1 Division of Outcomes and Value Research, John Theurer Cancer Center at Hackensack University Medical Center, 92 Second Street, Hackensack, NJ 07601 USA; 2 Hackensack Meridian Health, Hackensack, NJ USA; 3 Hackensack Meridian School of Medicine at Seton Hall University, Nutley, NJ USA; 4 Department of Biostatistics, Bioinformatics, and Biomathematics, Georgetown University, Washington, D.C., USA; 5 John Theurer Cancer Center at Hackensack University Medical Center, Hackensack, NJ USA; 6 COTA, Boston, MA USA; 7 Hackensack University Medical Center, Hackensack, NJ USA; 8 Bayshore Medical Center, Holmdel, NJ USA; 9 Jersey Shore University Medical Center, Neptune City, NJ USA; 10 JFK Medical Center, Edison, NJ USA; 11 Hackensack Meridian Mountainside Medical Center, Montclair, NJ USA; 12 Ocean Medical Center, Brick, NJ USA; 13 Palisades Medical Center, North Bergen, NJ USA; 14 Pascack Valley Medical Center, Westwood, NJ USA; 15 Raritan Bay Medical Center, Old Bridge, NJ USA; 16 Riverview Medical Center, Red Bank, NJ USA; 17 Southern Ocean Medical Center, Manahawkin, NJ USA

**Keywords:** Hydroxychloroquine, COVID-19, Outpatient

## Abstract

**Background:**

Hydroxychloroquine has not been associated with improved survival among hospitalized COVID-19 patients in the majority of observational studies and similarly was not identified as an effective prophylaxis following exposure in a prospective randomized trial. We aimed to explore the role of hydroxychloroquine therapy in mildly symptomatic patients diagnosed in the outpatient setting.

**Methods:**

We examined the association between outpatient hydroxychloroquine exposure and the subsequent progression of disease among mildly symptomatic non-hospitalized patients with documented SARS-CoV-2 infection. The primary outcome assessed was requirement of hospitalization. Data was obtained from a retrospective review of electronic health records within a New Jersey USA multi-hospital network. We compared outcomes in patients who received hydroxychloroquine with those who did not applying a multivariable logistic model with propensity matching.

**Results:**

Among 1274 outpatients with documented SARS-CoV-2 infection 7.6% were prescribed hydroxychloroquine. In a 1067 patient propensity matched cohort, 21.6% with outpatient exposure to hydroxychloroquine were hospitalized, and 31.4% without exposure were hospitalized. In the primary multivariable logistic regression analysis with propensity matching there was an association between exposure to hydroxychloroquine and a decreased rate of hospitalization from COVID-19 (OR 0.53; 95% CI, 0.29, 0.95). Sensitivity analyses revealed similar associations. QTc prolongation events occurred in 2% of patients prescribed hydroxychloroquine with no reported arrhythmia events among those with data available.

**Conclusions:**

In this retrospective observational study of SARS-CoV-2 infected non-hospitalized patients hydroxychloroquine exposure was associated with a decreased rate of subsequent hospitalization. Additional exploration of hydroxychloroquine in this mildly symptomatic outpatient population is warranted.

**Supplementary Information:**

The online version contains supplementary material available at 10.1186/s12879-021-05773-w.

## Background

The majority of infections with SARS-CoV-2 result in mildly symptomatic or asymptomatic illnesses that can be managed in outpatient settings. However, progression of the COVID-19 illness may result in significant morbidity and mortality requiring hospitalization and consumption of healthcare resources. To date, there are no treatments endorsed by the World Health Organization or Infectious Disease Societies of America for outpatient management of early disease [1, 2]. In New Jersey, an early COVID-19 epicenter in the United States, approximately 11% of positive cases required hospitalization (216 per 100,000 population) from March to July, 2020 [3]. As testing availability has increased and testing practices have broadened to include mildly symptomatic and asymptomatic individuals, the Centers for Disease Control and Prevention has reported a United States national cumulative COVID-19 hospitalization rate of 243.8 per 100,000 individuals [4].

Hydroxychloroquine, an antimalarial agent with antiviral and anti-inflammatory properties, has been touted as a potential therapy for COVID-19 [5]. Among hospitalized COVID-19 patients, observational studies have noted that hydroxychloroquine exposure has not been associated with a reduction in the risk of death [6–9]. A recent observational study from Michigan, however, reported improved survival when hydroxychloroquine was administered within 2 days of hospitalization [10]. When used as post-exposure prophylaxis within 4 days after moderate or high risk exposure, a prospective randomized trial found that hydroxychloroquine failed to prevent illness compatible with Covid-19 or confirmed infection [11].

Given that the majority of SARS-CoV-2 infected patients are mildly symptomatic and are managed in the outpatient setting, it remains important to explore whether early administration of hydroxychloroquine could delay progression to more severe illness requiring hospitalization. A trial from Spain randomized younger (mean age 41.6 years) mildly symptomatic outpatients to a 7-day course of hydroxychloroquine or observation, reporting no significant reductions in mean viral load or reduction in hospitalization rate (7.1% control versus 5.9% hydroxychloroquine) [12]. A second randomized study enrolled 491 USA and Canadian subjects via the internet, of whom 34% had virology confirmed infection. Although the overall hospitalization rate was only 3.2% within the population participating in the study (median age 40), more patients receiving placebo (4.7%) compared to hydroxychloroquine (1.9%) required hospitalization [13]. A Brazilian study of 636 symptomatic, but virology unconfirmed patients treated by telemedicine at home, also noted a reduction in hospitalization rate (5.4% vs 1.9%), with the greatest reductions occurring among the patients who started hydroxychloroquine therapy within the first 7 days of symptoms [14]. A small French report noted a reduction in symptoms with early therapy compared to observation [15]. Finally, a German report of 141 outpatients, when compared to cases in the community, noted a decrease in hospitalization rate (2.8% vs 15.4%) with a combination of hydroxychloroquine, azithromycin and zinc [16]. In summary, the majority of studies, although underpowered to show differences, are all directionally in favor of a reduced hospitalization rate with early outpatient treatment.

Understanding the limitations of observational studies, but with the urgency for evaluating potential therapeutic approaches during the current COVID-19 pandemic, our hospital spanning New Jersey USA established an observational database utilizing an integrated electronic health record (EHR) system (EPIC; Verona, WI) [17–20]. In this multi-center observational cohort study we report progression from mildly symptomatic SARS-CoV-2 infection diagnosed as an outpatient progressing to subsequent need for inpatient hospitalization according to outpatient exposure to hydroxychloroquine.

## Methods

### Study design and cohort selection

This retrospective, observational, multicenter cohort study within the Hackensack Meridian Health network (HMH) utilized EHR-derived data of patients with documented SARS-CoV-2 infection who received care initially within an outpatient setting. Our primary objective was to evaluate the association between hydroxychloroquine exposure and subsequent need for hospitalization in a population of patients with documented SARS-CoV-2 infection diagnosed in the outpatient setting.

Database inclusion and exclusion criteria for this review: 1) Positive SARS-CoV-2 diagnosis by reverse-transcriptase polymerase chain reaction, 2) Outpatient status (includes emergency room diagnosis without immediate hospitalization on the same day) at an HMH outpatient facility between March 1, 2020 until April 22, 2020. Follow-up continued through May 22, 2020.

Institutional Review Board (IRB) approval was obtained for access to the prospective observational database, under Hackensack Meridian Health IRB Study# Pro2020–0342. The requirement for patient informed consent (verbal or written) was waived by the IRB as this project represented a non-interventional study utilizing routinely collected data for secondary research purposes.

### Data sources

We collected data from HMH’s EHR (Epic) which is utilized throughout the network. Outpatients treated at a network related facility were flagged by the EHR if SARS-CoV-2 polymerase chain reaction tests were positive. These EHR-generated reports served as our eligible cohort sample. Demographic, clinical characteristics, treatments, and outcomes were manually abstracted by research nurses and physicians from the John Theurer Cancer Center at Hackensack University Medical Center. Assignment of patients to our data team occurred in real-time but was not randomized. To reduce sampling bias the final cohort included 100% of outpatients by April 22, 2020 as noted on the EHR-generated reports. Data abstracted by the team were entered utilizing Research Electronic Data Capture (REDCap). Quality control was performed by physicians (AI, SLG) overseeing nurse or physician abstraction. It should be also noted that data abstracted for this project, specifically lab data and hospitalization data, were also used in two other observational cohort studies on the effect of inpatient hydroxychloroquine and tocilizumab on COVID-19 outcomes [6, 21].

Demographic information was collected by an electronic face-sheet. Comorbidities were defined as diagnosed prior to hospitalization for COVID-19. If not listed in the patient’s record comorbidities were recorded as absent.

### Exposure

For hydroxychloroquine, exposure was defined as a prescription written for the drug as found in the EHR, by documentation in a provider note or in the medication section of the chart. No confirmation of prescription fill or adherence to the medication regimen was attempted. If no evidence of administration of the drug was found, this was recorded as not having received the drug. Hydroxychloroquine exposure, for the purpose of this study, was limited to initiation of treatment in the outpatient setting. Patients who did not have a prehospital exposure, who was subsequently admitted to a hospital, and then received first dose of hydroxychloroquine in the inpatient setting were counted as having no outpatient exposure to hydroxychloroquine.

### Outcome measures

The primary outcome measurement was subsequent need for hospitalization with follow-up until May 22, 2020. Hospitalization was identified on EHR review which includes the 13-hospitals within the Hackensack Meridian Health network. The EPIC system also notifies a limited number of participating hospitals outside the network (Epic Care-Everywhere). No attempt to contact the patient to confirm hospitalization outside the network was permitted or performed. Among patients who were hospitalized, the time from date of diagnosis to hospitalization and the requirement for intensive unit care level support or death was also collected. Safety events including discontinuation due to QTc prolongation or arrhythmia incidence after hydroxychloroquine exposure were recorded as per chart review.

Exploratory outcomes included the effect of outpatient hydroxychloroquine exposure on elderly patients over age 65, on patients with more than 2 days of self-reported symptoms, and on patients with at least one reported symptom of fever, shortness of breath, or cough.

### Statistical analysis

Demographic and clinical parameters of hydroxychloroquine treatment were summarized using median (Q1-Q3) for continuous variables and frequency (percentages) for categorical variables. The differences in the median/distributions of demographic and clinical parameters between the hydroxychloroquine treated and untreated (no hydroxychloroquine) groups were compared using Mood’s median test for continuous variables and Fisher’s exact test or Pearson’s chi-squared test for categorical variables. The comparator group in both the unmatched and propensity matched cohorts included only patients who did not receive hydroxychloroquine.

Multivariable adjusted logistic regression models were fitted to estimate the association between hydroxychloroquine exposure and the need for subsequent hospitalization using clinically likely confounders including age, gender, cancer, hypertension, COPD/asthma, diabetes, fever, cough, shortness of breath, and qSOFA score. When the model goodness-of-fit was not satisfied, we further reduced the aforementioned confounders using the stepwise variable selection and the lasso variable selection [22]. The odds ratios (OR) and their 95% confidence intervals were summarized.

To reduce the confounding effects secondary to imbalances in receiving hydroxychloroquine treatment inherent to a retrospective cohort study, we employed propensity-score matching. First, we calculated a propensity score (PS) of receiving hydroxychloroquine treatment for each patient using multivariable logistic regression via adjusting for the aforementioned set of confounder variables except time to hydroxychloroquine treatment. Goodness-of-fit of the multivariable logistic model was examined using the Hosmer-Lemeshow test. We then employed a nonparametric nearest neighbor matching of propensity scores to generate a matched cohort in a 1:10 ratio to pair a patient with hydroxychloroquine treatment to ten patients without hydroxychloroquine treatment (*MatchIt* Package in R) [22, 23].

With the propensity matched cohort, we repeated the adjusted logistic model with the propensity matched set similar to the unmatched analyses. Sensitivity analyses for confounders were conducted by including the propensity score as a covariate in the unmatched model and by including informative confounders chosen by stepwise selection. Missing data in categorical covariates were coded as a missing data category and were included in the all analyses. Completely observed data by excluding patients with missing covariates were also examined summarized in Supplementary Tables (see Additional file 1). The Kaplan-Meier method and log-rank test were performed to evaluate and compare the median time from date of diagnosis to hospitalization between the hydroxychloroquine treated and untreated groups. Furthermore, we performed an exploratory analysis from time of symptom onset to date of first dose of hydroxychloroquine. A cut-off of less than 2 days from time of symptom onset was used for a logistic regression analysis comparing those with early disease versus later as there appeared to be a stronger benefit to early administration of hydroxychloroquine [24]. Statistical significance was determined when two-sided *p*-value< 0.05. Subgroup analyses were performed exploratory and thus multiple-test correction was not applied. All statistical analyses were conducted using R software (ver. 3.4., R Project for Statistical Computing).

## Results

### Characterization of the study cohort

**Fig. 1:**
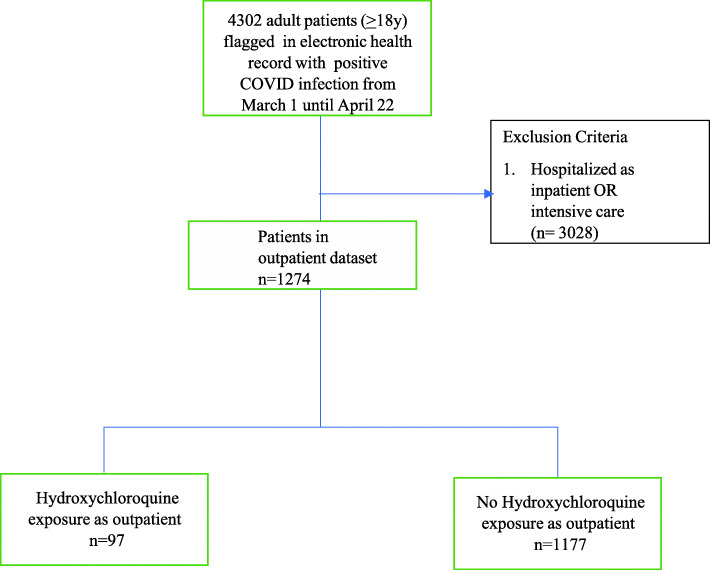
Cohort Selection Flow Diagram. Flow diagram of patient sampling strategy of non-hospitalized COVID-19 patients in Hackensack Meridian Health Network. Follow up occurred until May 22

There were 4302 patients flagged in the EHR with polymerase chain reaction confirmed infection with SARS-CoV-2. 1274 (30%) patients were evaluated and treated in the outpatient setting prior to any COVID-19 related hospitalization. Ninety-seven patients (7.6%) received prescriptions for hydroxychloroquine or had notation of an outpatient exposure to hydroxychloroquine (Fig. 1). 86 (87%) patients were prescribed 400 mg twice daily on day 1, and 400 mg daily on days 2–5, with the remaining were prescribed 200 mg three times a day (*n* = 6) or other (*n* = 5). The median duration of intended therapy prescribed was 5 days (IQR 4–5).

Given potential imbalances in treatment allocation due to the observational nature of the study a propensity matched sample was constructed consisting of 1067 patients in total (97 with hydroxychloroquine exposure and 970 without). The distribution of baseline characteristics is shown in Table 1. In the unmatched cohort patients exposed to hydroxychloroquine were more likely to have comorbid conditions. The propensity matched cohorts were well balanced except for an excess of cancer history and a trend towards older age in the hydroxychloroquine cohort.

**Table 1:** Baseline characteristics and outcomes

Characteristics	Unmatched Patients (***N*** = 1274)			Propensity-score-Matched Patients (***N*** = 1067)		
	No HCQ (***n*** = 1177)	HCQ (***n*** = 97)	***P***-value	No HCQ (***n*** = 970)	HCQ (***n*** = 97)	***P***-value
Age – median (IQR)	54 (40,64)	57 (44,65)	0.092	54 (40,65)	57 (44,65)	0.055
Gender, n(%)						
Female	583 (49.5)	56 (57.7)	0.148	531 (54.7)	56 (57.7)	0.647
Male	594 (50.5)	41 (42.3)		439 (45.3)	41 (42.3)	
Race/Ethnicity, n(%)						
African American	89 (7.6)	6 (6.2)	0.302	80 (8.2)	6 (6.2)	0.238
Asian	41 (3.5)	1 (1.0)		38 (3.9)	1 (1.0)	
Caucasian	602 (51.1)	57 (58.8)		497 (51.2)	57 (58.8)	
Hispanic	183 (15.5)	15 (15.5)		160 (16.5)	15 (15.5)	
Other	145 (12.3)	6 (6.2)		111 (11.4)	6 (6.2)	
Missing	117 (9.9)	12 (12.4)		84 (8.7)	12 (12.4)	
Nursing Home/Rehab resident, n(%)						
Yes	98 (8.3)	10 (10.3)	0.715	92 (9.5)	10 (10.3)	0.888
No	989 (84.0)	81 (83.5)		827 (85.3)	81 (83.5)	
Missing	90 (7.6)	6 (6.2)		51 (5.3)	6 (6.2)	
Academic vs Community						
Community	935 (79.4)	77 (79.4)	> 0.999	768 (79.2)	77 (79.4)	> 0.999
Academic	242 (20.6)	20 (20.6)		202 (20.8)	20 (20.6)	
Former or Current Smoker						
Yes	246 (20.9)	24 (24.7)	0.223	206 (21.2)	24 (24.7)	0.672
No	779 (66.2)	66 (68.0)		679 (70.0)	66 (68.0)	
Missing	152 (12.9)	7 (7.2)		85 (8.8)	7 (7.2)	
**Comorbidities, n(%)**						
Comorbidity Count^a^						
0	524 (44.5)	27 (27.8)	**0.005**	364 (37.5)	27 (27.8)	0.157
1	291 (24.7)	25 (25.8)		258 (26.6)	25 (25.8)	
2	197 (16.7)	23 (23.7)		191 (19.7)	23 (23.7)	
*≥* 3	165 (14.0)	22 (22.7)		157 (16.2)	22 (22.7)	
Diabetes, n(%)						
Yes	178 (15.1)	17 (17.5)	0.077	167 (17.2)	17 (17.5)	0.951
No	830 (70.5)	74 (76.3)		750 (77.3)	74 (76.3)	
Missing	169 (14.4)	6 (6.2)		53 (5.5)	6 (6.2)	
COPD/asthma, n(%)						
Yes	141 (12.0)	16 (16.5)	**0.040**	135 (13.9)	16 (16.5)	0.777
No	861 (73.2)	75 (77.3)		777 (80.1)	75 (77.3)	
Missing	175 (14.9)	6 (6.2)		58 (6.0)	6 (6.2)	
Hypertension, n(%)						
Yes	399 (33.9)	42 (43.3)	**0.013**	385 (39.7)	42 (43.3)	0.777
No	615 (52.3)	51 (52.6)		541 (55.8)	51 (52.6)	
Missing	163 (13.8)	4 (4.1)		44 (4.5)	4 (4.1)	
Coronary Disease, n(%)						
Yes	80 (6.8)	9 (9.3)	**0.035**	75 (7.7)	9 (9.3)	0.865
No	913 (77.6)	82 (84.5)		834 (86.0)	82 (84.5)	
Missing	184 (15.6)	6 (6.2)		61 (6.3)	6 (6.2)	
Stroke, n(%)						
Yes	24 (2.0)	4 (4.1)	**0.014**	23 (2.4)	4 (4.1)	0.480
No	974 (82.8)	87 (89.7)		889 (91.6)	87 (89.7)	
Missing	179 (15.2)	6 (6.2)		58 (6.0)	6 (6.2)	
Heart Failure, n(%)						
Yes	37 (3.1)	4 (4.1)	0.056	36 (3.7)	4 (4.1)	0.821
No	958 (81.4)	86 (88.7)		874 (90.1)	86 (88.7)	
Missing	182 (15.5)	7 (7.2)		60 (6.2)	7 (7.2)	
Arrhythmia, n(%)						
Yes	41 (3.5)	4 (4.1)	0.044	39 (4.0)	4 (4.1)	> 0.999
No	953 (81.0)	87 (89.7)		870 (89.7)	87 (89.7)	
Missing	183 (15.5)	6 (6.2)		61 (6.3)	6 (6.2)	
Cancer, n(%)						
Yes	87 (7.4)	20 (20.6)	**< 0.001**	80 (8.2)	20 (20.6)	**< 0.001**
No	914 (77.7)	72 (74.2)		833 (85.9)	72 (74.2)	
Missing	176 (15.0)	5 (5.2)		57 (5.9)	5 (5.2)	
Renal Failure, n(%)						
Yes	26 (2.2)	0 (0)	**0.010**	24 (2.5)	0 (0)	0.350
No	969 (82.3)	91 (93.8)		884 (91.1)	91 (93.8)	
Missing	182 (15.5)	6 (6.2)		62 (6.4)	6 (6.2)	
Rheumatologic Disorder, n(%)						
Yes	23 (2.0)	4 (4.1)	**0.020**	22(2.3)	4(4.1)	0.426
No	967 (82.2)	86 (88.7)		883 (91.0)	86 (88.7)	
Missing	187 (15.9)	7 (7.2)		65 (6.7)	7 (7.2)	
Obesity (BMI > 30), n(%)						
Yes	249 (21.2)	23 (23.7)	0.652	223 (23.0)	23 (23.7)	0.603
No	262 (22.3)	18 (18.6)		223 (23.0)	18 (18.6)	
Missing	666 (56.6)	56 (57.7)		524 (54.0)	56 (57.7)	
**Presenting Symptoms, n (%)**						
Fever, n(%)						
Yes	606 (51.5)	58 (59.8)	0.142	536 (55.3)	58 (59.8)	0.453
No	571 (48.5)	39 (40.2)		434 (44.7)	39 (40.2)	
Cough, n(%)						
Yes	642 (54.5)	54 (55.7)	0.914	531 (54.7)	54 (55.7)	0.946
No	535 (45.5)	43 (44.3)		439 (45.3)	43 (44.3)	
Shortness of Breath, n(%)						
Yes	392 (33.3)	35 (36.1)	0.656	345 (35.6)	35 (36.1)	> 0.999
No	785 (66.7)	62 (63.9)		625 (64.4)	62 (63.9)	
GI, n(%)						
Yes	172 (14.6)	12 (12.4)	0.650	141 (14.5)	12 (12.4)	0.669
No	1005 (85.4)	85 (87.6)		829 (85.5)	85 (87.6)	
Altered Mental State, n(%)						
Yes	38 (3.2)	3 (3.1)	> 0.999	36 (3.7)	3 (3.1)	> 0.999
No	1139 (96.8)	94 (96.9)		934 (96.3)	94 (96.9)	
Lack of taste or smell, n(%)						
Yes	65 (5.5)	6 (6.2)	0.965	56 (5.8)	6 (6.2)	> 0.999
No	1112 (94.5)	91 (93.8)		914 (94.2)	91 (93.8)	
**Disease severity, n(%)**						
Oxygenation *<* 94						
Yes	135 (11.5)	11 (11.3)	0.620	125 (12.9)	11 (11.3)	0.396
No	407 (34.6)	29 (29.9)		345 (35.6)	29 (29.9)	
Missing	635 (54.0)	57 (58.8)		500 (51.5)	57 (58.8)	
qSOFA Score						
0	416 (35.3)	31 (32.0)	0.575	343 (35.4)	31 (32.0)	0.577
*≥* 1	761 (64.7)	66 (68.0)		627 (64.6)	66 (68.0)	
**Initial laboratory test**						
Ferritin (IQR)	719.35 (298.18,1347.53)	537.37 (316.05,1240,87)	0.22	719.35 (291.78,1347.18)	537.37 (316.05,1240,87)	0.216
CRP (IQR)	7.79 (3.92,15.04)	16.64 (4.40,23.13)	0.340	9.51 (4.48,15.59)	16.64 (4.40,23.13)	0.241
IL-6 (IQR)	14 (5,36)	10 (6,10)	0.361	15 (5,39)	10 (6,10)	0.360
D-dimer (IQR)	0.98 (0.56,1.82)	0.93 (0.53,1.54)	> 0.999	1.01 (0.55,1.86)	0.93 (0.53,1.54)	> 0.999
Neutrophil (IQR)	4.5 (3.2,7.3)	3.8 (2.6,6.1)	0.473	4.6 (3.3,7.4)	3.8 (2.6,6.1)	0.366
Lymphocyte (IQR)	0.99 (0.68,1.40)	0.83 (0.7,1.1)	0.369	0.96 (0.66,1.40)	0.83 (0.7,1.1)	0.366
Neutrophil / Lymphocyte						
< 4.85	188 (16.0)	19 (19.6)	0.505	161 (16.6)	19 (19.6)	
≥ 4.85	159 (13.5)	15 (15.5)		146 (15.1)	15 (15.5)	0.731
Missing	830 (70.5)	63 (64.9)		663 (68.4)	63 (64.9)	
**Time**						
Follow-up time	38 (6,46)	42 (31,46)	0.091	38 (6,46)	42 (31,46)	0.070
# of days prior to diagnosis	5 (3,7)	5 (2,8)	0.609	5 (3,7)	5 (2,8)	0.604
**Outcomes, n(%)**						
Hospitalization						
Yes	350 (29.7)	21 (21.6)	0.117	305 (31.4)	21 (21.6)	0.060
No	827 (70.3)	76 (78.4)		665 (68.6)	76 (78.4)	
ICU admission						
Yes	46 (4.0)	3 (3.1)	> 0.999	42 (4.3)	3 (3.1)	0.791
No	1130 (96.0)	94 (96.9)		928 (95.7)	94 (96.9)	
Death						
Yes	47 (4)	2 (2.1)	0.578	44 (4.5)	2 (2.1)	0.427
No	1129 (96)	95 (97.9)		926 (95.5)	95 (97.9)	
**AE, n(%)**						
QT prolongation^b^						
Yes	3 (0.3)	2 (2.1)	0.049	3 (0.3)	2 (2.1)	0.068
No	1174 (99.7)	95 (97.9)		967 (99.7)	95 (97.9)	
Arrhythmia event^c^						
Yes	1 (0.1)	0 (0.01)	> 0.999	1 (0.1)	0 (0)	> 0.999
No	1176 (99.9)	97 (100.0)		969 (99.9)	97 (100.0)	

(1) Comorbidity count^a^: Diabetes, COPD/Asthma, Hypertension, Coronary Disease, Cerebrovascular disease, Heart Failure, Arrhythmia, Cancer Renal failure, Rheumatologic, disorder, and Obesity

(2) 10 Variables are used to do the matching: Age, gender, Cancer, Hypertension, COPD/Asthma, Diabetes, Fever, Cough, Shortness of Breath, SOFA Score

(3) If the variable tested is continuous, then a Mood’s median test is performed to compare medians of samples

If the variable tested is categorical, then a Pearson’s Chi-squared test or Fisher’s Exact test is performed

^b^QT prolongation that led to discontinuation of hydroxychloroquine. Those who did not receive outpatient hydroxychloroquine may have been exposed to hydroxychloroquine if hospitalized, and thus patients in the non-exposure group may have this adverse event reported

^c^Arrhythmia event recorded after COVID-19 diagnosis

In the propensity matched cohort 3 (3.1%) patients with outpatient exposure to hydroxychloroquine subsequently required ICU level support and 42 (4.3%) patients without exposure required ICU care. Ultimately, 2 (2.1%) patients with outpatient exposure to hydroxychloroquine died from COVID-19 related disease and 44 (4.5%) of patients without exposure died (Table 1).

### Primary study endpoints

**Fig. 2:**
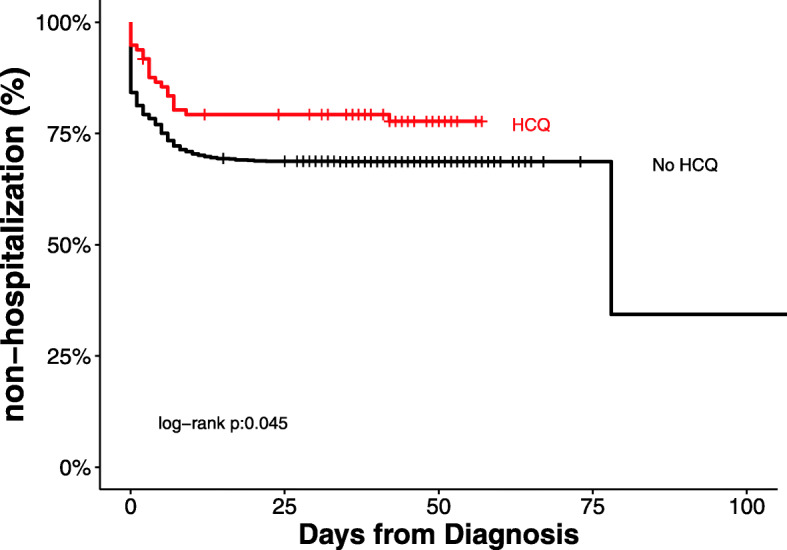
Hospitalization according to Hydroxychloroquine Exposure from Date of Confirmed SARS-CoV-2 Infection. Cumulative prevalence of hospitalization among mildly symptomatic COVID-19 patients according to outpatient exposure to hydroxychloroquine from date of polymerase chain reaction confirmed infection with SARS-CoV-2 in propensity matched cohort. HCQ = hydroxychloroquine

Among the 1067 outpatients in the propensity matched cohort, with a median of 39 days (IQR 6,46) follow-up, a total of 326 (30.6%) patients required subsequent hospitalization. Three hundred and five (31.4%) patients with no outpatient exposure to hydroxychloroquine were hospitalized and 21 (21.6%) of patients with exposure to hydroxychloroquine were hospitalized. Figure 2 shows the cumulative prevalence of hospitalization from date of diagnosis according to outpatient hydroxychloroquine exposure (log-rank *p* = 0.045). The cumulative prevalence of hospitalization from the self-reported date of onset of symptoms is shown in Supplementary Figure 1 (log-rank *p* = 0.036, see Additional file 1). 46 (4%) patients with no outpatient exposure required ICU care compared to 3 (3.1%) patients who had outpatient exposure to hydroxychloroquine. 47 (4%) patients with no outpatient exposure died compared to 2 (2%) patients with outpatient exposure to hydroxychloroquine. In patients prescribed hydroxychloroquine as an outpatient for whom follow-up electrocardiographic data were available, QTc prolongation events, defined as discontinuation due to physician discretion, occurred in 2 (2%) of patients, and arrhythmia events after hydroxychloroquine exposure were noted in no patients (Table 1).

In the primary multivariable logistic regression analysis with propensity matching there was an association between exposure to hydroxychloroquine and a reduced rate of hospitalization related to progressive COVID-19 illness (OR 0.53; 95% CI, 0.29, 0.95, unadjusted OR 0.60; 95% CI, 0.36, 0.98) (Table 2). Sensitivity analyses using stepwise (AIC based) variable and Lasso selection yielded similar results in the propensity matched cohorts (Supplementary tables 1-2, see Additional file 1), and the significant association was also identified in the unmatched cohort (Supplementary tables 3-6, see Additional file 1). Sensitivity analyses by excluding missing data also yielded similar results (Supplementary tables 1-6, see Additional file 1).

**Table 2:** Matched multivariable adjusted/unadjusted logistic regression models for hospitalization (sample size = 1067)

	Multivariable Adjusted			Unadjusted		
	Estimated OR	OR 95% CI	***P*** value	Estimated OR	OR 95% CI	***P*** value
HCQ, yes/no	0.535	(0.291,0.949)	0.038	0.602	(0.356,0.977)	0.048
Age	1.023	(1.012,1.034)	< 0.001	1.021	(1.014,1.029)	< 0.001
Gender, male/female	1.316	(0.957,1.81)	0.091	1.542	(1.187,2.005)	0.001
Diabetes, yes/no	1.255	(0.829,1.895)	0.281	1.653	(1.187,2.295)	0.003
Hypertension yes/no	0.941	(0.644,1.372)	0.753	1.314	(1.007,1.714)	0.044
COPD/Asthma, yes/no	0.718	(0.453,1.126)	0.154	1.093	(0.753,1.571)	0.633
Cancer, yes/no	1.045	(0.607,1.785)	0.872	1.458	(0.948,2.220)	0.081
**Presenting Symptoms, n (%)**						
Fever, yes/no	1.265	(0.866,1.85)	0.224	1.886	(1.441,2.478)	< 0.001
Cough, yes/no	0.853	(0.573,1.264)	0.43	1.670	(1.280,2.187)	< 0.001
Shortness of Breath, yes/no	6.113	(4.307,8.765)	< 0.001	6.210	(4.683,8.278)	< 0.001
**Disease severity, n (%)**						
qSOFA Score, 1/0	0.193	(0.139,0.265)	< 0.001	0.178	(0.134,0.236)	< 0.001

Test of model goodness of fit shows a good fit with *p*-value = 0.094 (g = 13) for the multivariable adjusted logistic regression model

### Exploratory study endpoints

In an exploratory analysis we examined a subgroup of 749 outpatients in the propensity matched cohort who self-reported at least one major symptom of fever, cough or shortness of breath at the time of their time of SARS-CoV-2 diagnosis. In this subgroup 69 (9.2%) patients received hydroxychloroquine prescriptions and 680 (90.8%) patients did not. There were fewer hospitalizations in the hydroxychloroquine cohort (19 patients, 27.5%) compared to individuals with no exposure (259 patients, 38.1%). In the multivariable logistic regression analysis of these symptomatic patients, there was no significant association between hydroxychloroquine exposure and subsequent need for hospitalization (OR 0.74, 95% CI, 0.39, 1.37) (Supplementary table 7, Supplementary figure 2, see Additional file 1).

Given the strong association between advanced age and subsequent hospitalization requirement in both the unmatched and propensity matched analyses, an additional analysis was conducted on the interaction between age and hydroxychloroquine exposure. Restricting the multivariable logistic regression model to the 282 persons age 65 years or greater resulted in a non-significant odds reduction of hospitalization (OR 0.49, 95% CI 0.17, 1.32). Similar directional trends were seen on sensitivity analyses in this elderly cohort (Supplementary table 8A-C, see Additional file 1).

A final subgroup analysis was conducted in patients who were exposed to outpatient hydroxychloroquine according to duration of symptoms, more than 2 days of self-reported symptoms compared to 2 days or less. A univariate logistic regression analysis did not show a significant association with hospitalization (OR 3.43, 95% CI 0.57, 66) (Supplementary table 9, see Additional file 1).

## Discussion

In this multicenter retrospective observational cohort study of mildly symptomatic outpatients with polymerase chain reaction documented SARS-CoV-2 infection, we noted an association (OR 0.53; 95% CI, 0.29, 0.95) between outpatient exposure to hydroxychloroquine and a reduction in subsequent need for hospitalization. Safety events, defined as QT prolongation or arrhythmia occurrence, were minimal, occurring in 2 and 0% of patients. As the majority of COVID-19 patients are mildly symptomatic and treated in outpatient settings, our findings justify further exploration of hydroxychloroquine during this pandemic in this population. It should be noted a recent observational cohort study from Brazil found a similar reduction in hospitalization if outpatient hydroxychloroquine was given [25]. If the findings are confirmed, early hydroxychloroquine therapy to a broad outpatient population could have important implications for reducing limited healthcare resources. The economic impact on healthcare might also be significant as the financial cost of a short course of hydroxychloroquine to a large population would be easily recouped by even a modest reduction in hospitalizations. The ease of oral administration also has added benefits compared to intravenous COVID-19 outpatient therapies recently given FDA emergency use approval [26].

Our findings in the outpatient setting are in conflict with prior observational studies conducted among hospitalized patients potentially highlighting differences in effect based on the severity of disease [27]. Following an initial infection by SARS-CoV-2 resulting in attack of alveolar epithelial cells patients may develop a hyper-inflammatory state characterized by activation of the innate immune system and release of pro-inflammatory cytokines and chemokines. Patients who experience this ‘cytokine storm’ progress rapidly to respiratory failure and multi-organ failure [28–31]. In these hospitalized patients the weak anti-inflammatory effects of hydroxychloroquine may be insufficient to block the cytokine cascade, whereas more potent immunosuppressive agents such as dexamethasone and tocilizumab have been associated with beneficial effects [21, 32, 33].

By contrast, hydroxychloroquine has anti-viral effects, decreasing SARS-CoV-2 viral load, and thus may be more suited in preventing the significant tissue damage needed to incite the hyper-inflammatory state [5, 34]. This would position hydroxychloroquine earlier in the clinical course, at the time of early infection, prior to hospitalization need [35].

As noted above, several recent studies have attempted to explore the role of hydroxychloroquine earlier in the clinical course of COVID-19 [12–16]. However, given enrollment of generally younger patients with low baseline rates of hospitalization, these studies appear under-powered to demonstrate meaningful effects. For example, the recent Spanish randomized trial explored early hydroxychloroquine use, at a median time from symptom onset of 3 days, in the outpatient setting [12]. While the study did not find a significant decrease in mean viral load up to 7 days after treatment, the investigators reported lower hospitalization rates in the population treated with hydroxychloroquine. Similar non-statistical directional reductions were noted in the other studies. To increase power and synthesize the current landscape, a meta-analysis of outpatient randomized controlled studies was conducted, examining prevention of COVID-19 in 2 trials, and reduced hospitalization or death 3 trials. Using a composite endpoint of reduced risk of infection or risk of hospitalization or death, Ladapo et al. identified a significant benefit with early use of hydroxychloroquine among outpatients infected with SARS-CoV-2 [36]. Thus, the potential benefit of hydroxychloroquine in the early management of outpatients should be of great interest and the subject of continued rigorous investigation.

We defined exposure to hydroxychloroquine based on documentation of a prescription being written, but confirmation of prescription fill or full adherence to the complete course was not ascertained, thus mimicking an intention-to-treat model. This limitation biased against finding a difference between cohorts, as non-adherent patients would be categorized within the hydroxychloroquine cohort even though in actuality, they did not have drug exposure. Thus, our reduction in hospitalization association may be an underestimate of the effect size, although without confirmation we acknowledge this is a major limitation. Conversely, it is possible that some outpatients received prescriptions for hydroxychloroquine outside the HMH network and were misclassified in the opposite direction, although this is less likely as patients underwent initial testing within our hospital network and would have been contacted by HMH personnel to discuss testing results and/or had notation of a prescription fill in the EPIC pharmacy section.

Our study was conducted early in the United States pandemic during a timeframe when testing for COVID-19 was largely limited to individuals with symptomatic disease. Thus, we suspect that those included in our observational cohort represent a bias towards more advanced disease with a higher likelihood of hospitalization. Indeed 30.6% of our cohort subsequently required hospital-based care, which is higher than current state and national hospitalization rates [3, 4]. Our findings need to be taken into context of current testing availability.

This observational study has several additional limitations. We recorded hospitalizations based on EHR documentation, but we have not accounted for hospitalizations outside the HMH network. Since the patients in our series received outpatient care at an HMH facility we believe that subsequent hospitalizations outside the network were minimal. Observational studies also cannot draw causal inferences given inherent known and unknown confounders. We attempted to adjust for known confounders using our propensity model approach but acknowledge we may not have captured all possible confounders. Misclassifications of the data are possible due to manual abstraction of EHR structured and unstructured data. Missing data, laboratory studies not obtained, and symptoms not reported or documented also limited our analyses. This especially affected our assessment of severity on presentation as we did not have inflammatory markers or imaging findings, which might have aided in triaging need for hospitalization or additional therapy [37]. Our study also focused on patients in New Jersey USA, limiting the applicability to other geographic regions with differing treatment and hospitalization algorithms. Lastly, we were limited by sample size, as we noted several non-significant trends in reduced hospitalizations in the elderly over age 65 (OR 0.49, 95% CI 0.17, 1.32) and in symptomatic patient (OR 0.74, 95% CI 0.39, 1.37) subgroups.

In conclusion, hydroxychloroquine exposure among outpatients with mildly symptomatic COVID-19 was associated with a reduction in hospitalization rates from disease progression in this multi-center observational cohort. Further external validation of this finding is required. Although use of hydroxychloroquine in this outpatient population outside the context of a clinical trial cannot be recommended, our study suggests that additional evaluations of hydroxychloroquine are needed in this mildly symptomatic SARS-CoV-2 infected population.

## Supplementary Information

**Additional file 1: Supplementary Figure 1.** Hospitalization according to Hydroxychloroquine Exposure from Self-Reported Onset of COVID-19 Symptoms. **Supplementary Table 1.** Matched multivariate logistic regression model with the stepwise (AIC based) variable selection procedure for hospitalization. **Supplementary Table 2.** Matched regression model with variables selected by Lasso. **Supplementary Table 3.** Unmatched multivariable logistic regression model for hospitalization. **Supplementary Table 4.** Unmatched multivariable logistic regression model with the stepwise (AIC based) variable selection procedure for hospitalization. **Supplementary Table 5.** Unmatched multivariable regression model with variables selected by Lasso. **Supplementary Table 6.** Unmatched multivariable logistic regression model with PS score for hospitalization. **Supplementary Table 7.** Multivariate logistic regression model for hospitalization in symptomatic subgroup. **Supplementary Figure 2.** Hospitalization according to Hydroxychloroquine Exposure Among Cohort with Fever, Cough or Shortness of Breath at Time of Evaluation. **Supplementary Table 8.** A-C Matched multivariate logistic regression models for hospitalization in age > 65. **Supplementary Table 9.** Univariate logistic regression in HCQ subgroup with symptoms > 2 days compared to < 2 days.

## Data Availability

The dataset and analysis supporting the conclusions of this article are available in the Synapse repository, unique DOI 10.7303/syn22909530 [38]. The website link is https://www.synapse.org/#!Synapse:syn22909530/files/. The files are restricted by copyright protection under Hackensack Meridian Health, who owns the data. Data may be made available from the authors upon reasonable request and with licensing permission from Hackensack Meridian Health.
