# Supplementary material for: Hydroxychloroquine in the treatment of outpatients with mildly symptomatic COVID-19: a multi-center observational study
Source: BMC Infect Dis. 2021 Jan 14;21:72. doi: 10.1186/s12879-021-05773-w (PMC7807228; doi:10.1186/s12879-021-05773-w)
Supplement: Supplementary file 1 — Additional file 1: Supplementary Figure 1. Hospitalization according to Hydroxychloroquine Exposure from Self-Reported Onset of COVID-19 Symptoms. Supplementary Table 1. Matched multivariate logistic regression model with the stepwise (AIC based) variable selection procedure for hospitalization. Supplementary Table 2. Matched regression model with variables selected by Lasso. Supplementary Table 3. Unmatched multivariable logistic regression model for hospitalization. Supplementary Table 4. Unmatched multivariable logistic regression model with the stepwise (AIC based) variable selection procedure for hospitalization. Supplementary Table 5. Unmatched multivariable regression model with variables selected by Lasso. Supplementary Table 6. Unmatched multivariable logistic regression model with PS score for hospitalization. Supplementary Table 7. Multivariate logistic regression model for hospitalization in symptomatic subgroup. Supplementary Figure 2. Hospitalization according to Hydroxychloroquine Exposure Among Cohort with Fever, Cough or Shortness of Breath at Time of Evaluation. Supplementary Table 8. A-C Matched multivariate logistic regression models for hospitalization in age > 65. Supplementary Table 9. Univariate logistic regression in HCQ subgroup with symptoms > 2 days compared to < 2 days. [file 12879_2021_5773_MOESM1_ESM.docx]

**Additional File 1**

**Table of Contents**

| **Page** | **Table/Figure** | **Title** |
| --- | --- | --- |
| 2 | Supplementary Figure 1 | Hospitalization according to Hydroxychloroquine Exposure from Self-Reported Onset of COVID-19 Symptoms |
| 3 | Supplementary Table 1 | Matched multivariate logistic regression model with the stepwise (AIC based) variable selection procedure for hospitalization |
| 4 | Supplementary Table 2 | Matched regression model with variables selected by Lasso |
| 5-6 | Supplementary Table 3 | Unmatched multivariable logistic regression model for hospitalization |
| 7 | Supplementary Table 4 | Unmatched multivariable logistic regression model with the stepwise (AIC based) variable selection procedure for hospitalization |
| 8 | Supplementary Table 5 | Unmatched multivariable regression model with variables selected by Lasso |
| 9 | Supplementary Table 6 | Unmatched multivariable logistic regression model with PS score for hospitalization |
| 10 | Supplementary Table 7 | Multivariate logistic regression model for hospitalization in symptomatic subgroup |
| 11 | Supplementary Figure 2 | Hospitalization according to Hydroxychloroquine Exposure Among Cohort with Fever, Cough or Shortness of Breath at Time of Evaluation |
| 12 | Supplementary table 8A-C | Matched multivariate logistic regression models for hospitalization in age >65 |
| 13 | Supplementary table 9 | Univariate logistic regression in HCQ subgroup with symptoms >2 days compared to <2 days |

Authors

Andrew Ip, M.D. M.S., Jaeil Ahn, Ph.D., Yizhao Zhou, M.S., Andre H. Goy, M.D., Eric Hansen, B.S. M.S., Andrew L Pecora, M.D., Brittany A Sinclaire, M.S., Urszula Bednarz, B.S CCRP, Michael Marafelias, B.S., Shivam Mathura, B.A., Ihor S Sawczuk, M.D., Joseph P. Underwood, M.D., David M. Walker, M.D., Rajiv Prasad, M.D., Robert L. Sweeney, D.O., Marie G. Ponce, M.D., Samuel La Capra, M.D., Frank J. Cunningham, MD, Arthur G. Calise, D.O., Bradley L. Pulver, M.D., Dominic Ruocco, M.D., Greggory E. Mojares, D.O., Michael P. Eagan, M.D., Kristy L. Ziontz, D.O., Paul Mastrokyriakos, D.O., and Stuart L Goldberg, M.D.

**Supplementary Figure 1**

**Hospitalization according to Hydroxychloroquine Exposure from Self-Reported Onset of COVID-19 Symptoms**

**Cumulative prevalence of hospitalization among mildly symptomatic COVID-19 patients according to outpatient exposure to hydroxychloroquine from onset of self-reported symptoms**

**HCQ=hydroxychloroquine.**

Supplementary Table 1

**Matched multivariate logistic regression model with the stepwise (AIC based) variable selection procedure for hospitalization (sample size=1067)**

|  | **Estimated OR** | **OR 95% CI** | **P value** |
| --- | --- | --- | --- |
| HCQ, yes/no | 0.546 | (0.3,0.96) | 0.040 |
| Age | 1.024 | (1.01,1.03) | <0.001 |
| Gender, male/female | 1.373 | (1.00,1.88) | 0.047 |
| COPD/Asthma, yes/no | 0.682 | (0.43,1.07) | 0.096 |
| **Presenting Symptoms, n (%)** |  |  |  |
| Shortness of Breath, yes/no | 6.309 | (4.59,8.74) | <0.001 |
| **Disease severity,**  **n (%)** |  |  |  |
| qSOFA Score, 1/0 | 0.187 | (0.14,0.26) | <0.001 |

Note : Test of model goodness of fit shows a good fit with p-value=0.344 (g=8)

Supplementary Table 1 – missing data excluded

**Matched multivariate logistic regression model with the stepwise (AIC based) variable selection procedure hospitalization excluding individuals with any missing values. (sample size=993)**

|  | **Estimated OR** | **OR 95% CI** | **P value** |
| --- | --- | --- | --- |
| HCQ, yes/no | 0.527 | (0.29,0.94) | 0.033 |
| Age | 1.023 | (1.01,1.03) | <0.001 |
| Gender, male/female | 1.281 | (0.93,1.76) | 0.125 |
| COPD/Asthma, yes/no | 0.673 | (0.43,1.05) | 0.087 |
| **Presenting Symptoms, n (%)** |  |  |  |
| Shortness of Breath, yes/no | 6.219 | (4.51,8.64) | <0.001 |
| **Disease severity,**  **n (%)** |  |  |  |
| qSOFA Score, 1/0 | 0.193 | (0.14,0.27) | <0.001 |

Note : Test of model goodness of fit shows a good fit with p-value=0.708 (g=8)

Supplementary Table 2

**Matched regression model with variables selected by Lasso (sample size=1067)**

|  | **Estimated OR** | **OR 95% CI** | **P value** |
| --- | --- | --- | --- |
| HCQ, yes/no | 0.544 | (0.30,0.96) | 0.039 |
| Age | 1.023 | (1.01,1.03) | <0.001 |
| Gender, male/female | 1.34 | (0.98,1.84) | 0.069 |
| Diabetes, yes/no | 1.227 | (0.82,1.82) | 0.314 |
| COPD/Asthma, yes/no | 0.709 | (0.45,1.11) | 0.138 |
| **Presenting Symptoms, n (%)** |  |  |  |
| Fever, yes/no | 1.196 | (0.85,1.69) | 0.31 |
| Shortness of Breath, yes/no | 5.961 | (4.28,8.38) | <0.001 |
| **Disease severity, n (%)** |  |  |  |
| qSOFA Score, 1/0 | 0.188 | (0.14,0.26) | <0.001 |

Note : Test of model goodness of fit shows a good fit with p-value=0.444 (g=10)

Supplementary Table 2 – missing data excluded

**Matched regression model with variables selected by Lasso excluding individuals with any missing values. (sample size=993)**

|  | **Estimated OR** | **OR 95% CI** | **P value** |
| --- | --- | --- | --- |
| HCQ, yes/no | 0.524 | (0.28,0.93) | 0.032 |
| Age | 1.023 | (1.01,1.03) | <0.001 |
| Gender, male/female | 1.23 | (0.89,1.69) | 0.206 |
| Diabetes, yes/no | 1.237 | (0.83,1.84) | 0.297 |
| COPD/Asthma, yes/no | 0.7 | (0.44,1.10) | 0.127 |
| **Presenting Symptoms, n (%)** |  |  |  |
| Fever, yes/no | 1.303 | (0.89,1.91) | 0.174 |
| Cough, yes/no | 0.818 | (0.55,1.22) | 0.323 |
| Shortness of Breath, yes/no | 6.18 | (4.35,8.88) | <0.001 |
| **Disease severity, n (%)** |  |  |  |
| qSOFA Score, 1/0 | 0.195 | (0.14,0.27) | <0.001 |

Note : Test of model goodness of fit shows a good fit with p-value=0.145 (g=11)

Supplementary Table 3

**Unmatched multivariable logistic regression model for hospitalization (sample size=1274)**

|  | **Multivariable adjusted** | | | **Unadjusted** | | |
| --- | --- | --- | --- | --- | --- | --- |
|  | **Estimated OR** | **OR 95% CI** | **P value** | **Estimated OR** | **OR 95% CI** | **P value** |
| HCQ, yes/no | 0.533 | (0.29,0.94) | 0.034 | 0.653 | (0.39,1.06) | 0.094 |
| Age | 1.02 | (1.01,1.03) | <0.001 | 1.019 | (1.01,1.03) | <0.001 |
| Gender, male/female | 1.361 | (1.01,1.83) | 0.042 | 1.445 | (1.13,1.84) | 0.003 |
| Diabetes, yes/no | 1.255 | (0.84,1.87) | 0.262 | 1.551 | (1.13,2.13) | 0.007 |
| Hypertension yes/no | 0.959 | (0.67,1.37) | 0.817 | 1.212 | (0.94,1.56) | 0.138 |
| COPD/Asthma, yes/no | 0.73 | (0.47,1.13) | 0.162 | 1.019 | (0.71,1.45) | 0.917 |
| Cancer, yes/no | 1.022 | (0.60,1.72) | 0.934 | 1.269 | (0.84,1.91) | 0.256 |
| **Presenting Symptoms, n (%)** |  |  |  |  |  |  |
| Fever, yes/no | 1.274 | (0.90,1.80) | 0.167 | 2.174 | (1.69,2.80) | <0.001 |
| Cough, yes/no | 0.847 | (0.59,1.22) | 0.371 | 1.981 | (1.54,2.55) | <0.001 |
| Shortness of Breath, yes/no | 5.707 | (4.13,7.96) | <0.001 | 6.171 | (4.75,8.05) | <0.001 |
| **Disease severity, n (%)** |  |  |  |  |  |  |
| qSOFA Score, 1/0 | 0.19 | (0.14,0.26) | <0.001 | 0.159 | (0.12,0.21) | <0.001 |

Note : Test of model goodness of fit shows a good fit with p-value=0.436 (g=13) ) for the multivariate logistic regression model. Neutrophil / Lymphocyte was not used due to its high missing frequency (70%).

**Unmatched multivariable logistic regression model for hospitalization excluding individuals with any missing values. (sample size=1077)**

|  | **Estimated OR** | **OR 95% CI** | **P value** |
| --- | --- | --- | --- |
| HCQ, yes/no | 0.51 | (0.28,0.91) | 0.026 |
| Age | 1.021 | (1.01,1.03) | <0.001 |
| Gender, male/female | 1.265 | (0.94,1.71) | 0.125 |
| Diabetes, yes/no | 1.246 | (0.83,1.86) | 0.281 |
| Hypertension yes/no | 0.938 | (0.66,1.34) | 0.728 |
| COPD/Asthma, yes/no | 0.713 | (0.45,1.11) | 0.137 |
| Cancer, yes/no | 1.08 | (0.64,1.82) | 0.775 |
| **Presenting Symptoms, n (%)** |  |  |  |
| Fever, yes/no | 1.287 | (0.91,1.82) | 0.156 |
| Cough, yes/no | 0.843 | (0.58,1.22) | 0.366 |
| Shortness of Breath, yes/no | 5.63 | (4.06,7.87) | <0.001 |
| **Disease severity, n (%)** |  |  |  |
| qSOFA Score, 1/0 | 0.202 | (0.149,0.27) | <0.001 |

Note : Test of model goodness of fit shows a good fit with p-value=0.375 (g=13) ) for the multivariate logistic regression model.

Supplementary Table 4

**Unmatched multivariable logistic regression model with the stepwise (AIC based) variable selection procedure for hospitalization (sample size=1274)**

|  | **Estimated OR** | **OR 95% CI** | **P value** |
| --- | --- | --- | --- |
| HCQ, yes/no | 0.552 | (0.31,0.96) | 0.042 |
| Age | 1.021 | (1.01,1.03) | <0.001 |
| Gender, male/female | 1.4 | (1.04,1.88) | 0.025 |
| COPD/Asthma, yes/no | 0.695 | (0.45,1.07) | 0.103 |
| **Presenting Symptoms, n (%)** |  |  |  |
| Shortness of Breath, yes/no | 5.827 | (4.33,7.90) | <0.001 |
| **Disease severity, n (%)** |  |  |  |
| qSOFA Score, 1/0 | 0.186 | (0.14,0.25) | <0.001 |

Note : Test of model goodness of fit shows a good fit with p-value=0.193 (g=8)

Supplementary Table 4 – missing data excluded

**Unmatched multivariable logistic regression model with the stepwise (AIC based) variable selection procedure for hospitalization excluding individuals with any missing values. (sample size=1077)**

|  | **Estimated OR** | **OR 95% CI** | **P value** |
| --- | --- | --- | --- |
| HCQ, yes/no | 0.523 | (0.29,0.92) | 0.029 |
| Age | 1.021 | (1.01,1.03) | <0.001 |
| Gender, male/female | 1.298 | (0.96,1.75) | 0.086 |
| COPD/Asthma, yes/no | 0.693 | (0.44,1.07) | 0.104 |
| **Presenting Symptoms, n (%)** |  |  |  |
| Shortness of Breath, yes/no | 5.700 | (4.21,7.76) | <0.001 |
| **Disease severity, n (%)** |  |  |  |
| qSOFA Score, 1/0 | 0.200 | (0.15,0.27) | <0.001 |

Note : Test of model goodness of fit shows a good fit with p-value=0.269 (g=8)

Supplementary Table 5

**Unmatched multivariable regression model with variables selected by Lasso (sample size=1274)**

|  | **Estimated OR** | **OR 95% CI** | **P value** |
| --- | --- | --- | --- |
| HCQ, yes/no | 0.536 | (0.30,0.94) | 0.033 |
| Age | 1.02 | (1.01,1.03) | <0.001 |
| Gender, male/female | 1.361 | (1.01,1.83) | 0.042 |
| Diabetes, yes/no | 1.246 | (0.85,1.82) | 0.259 |
| COPD/Asthma, yes/no | 0.729 | (0.47,1.13) | 0.159 |
| **Presenting Symptoms, n (%)** |  |  |  |
| Fever, yes/no | 1.28 | (0.91,1.81) | 0.159 |
| Cough, yes/no | 0.855 | (0.59,1.23) | 0.397 |
| Shortness of Breath, yes/no | 5.759 | (4.17,8.02) | <0.001 |
| **Disease severity, n (%)** |  |  |  |
| qSOFA Score, 1/0 | 0.188 | (0.14,0.25) | <0.001 |

Note : Test of model goodness of fit shows a good fit with p-value=0.156 (g=11)

Supplementary Table 5 – missing data excluded

**Unmatched multivariable regression model with variables selected by Lasso excluding individuals with any missing values. (sample size=1077)**

|  | **Estimated OR** | **OR 95% CI** | **P value** |
| --- | --- | --- | --- |
| HCQ, yes/no | 0.515 | (0.28,0.91) | 0.026 |
| Age | 1.02 | (1.01,1.03) | <0.001 |
| Gender, male/female | 1.267 | (0.94,1.71) | 0.123 |
| Diabetes, yes/no | 1.221 | (0.83,1.79) | 0.308 |
| COPD/Asthma, yes/no | 0.711 | (0.45,1.10) | 0.133 |
| **Presenting Symptoms, n (%)** |  |  |  |
| Fever, yes/no | 1.288 | (0.91,1.83) | 0.153 |
| Cough, yes/no | 0.843 | (0.58,1.22) | 0.364 |
| Shortness of Breath, yes/no | 5.647 | (4.08,7.89) | <0.001 |
| **Disease severity, n (%)** |  |  |  |
| qSOFA Score, 1/0 | 0.201 | (0.15,0.27) | <0.001 |

Note : Test of model goodness of fit shows a good fit with p-value=0.446 (g=11)

Supplementary Table 6

**Unmatched multivariable logistic regression model with PS score for hospitalization (sample size=1274)**

|  | **Estimated OR** | **OR 95% CI** | **P value** |
| --- | --- | --- | --- |
| HCQ, yes/no | 0.532 | (0.29,0.94) | 0.034 |
| Age | 1.02 | (1.01,1.03) | <0.001 |
| Gender, male/female | 1.204 | (0.73,1.94) | 0.456 |
| Diabetes, yes/no | 1.26 | (0.85,1.87) | 0.255 |
| Hypertension yes/no | 1.017 | (0.68,1.52) | 0.936 |
| COPD/Asthma, yes/no | 0.802 | (0.47,1.36) | 0.417 |
| Cancer, yes/no | 1.835 | (0.29,13.31) | 0.536 |
| **Presenting Symptoms, n (%)** |  |  |  |
| Fever, yes/no | 1.481 | (0.84,2.7) | 0.191 |
| Cough, yes/no | 0.792 | (0.52,1.20) | 0.279 |
| Shortness of Breath, yes/no | 5.738 | (4.15,8.00) | <0.001 |
| **Disease severity, n (%)** |  |  |  |
| qSOFA Score, 1/0 | 0.219 | (0.13,0.38) | <0.001 |
| PS score | 0.005 | (0,39274.80) | 0.537 |

Note : Test of model goodness of fit shows a good fit with p-value=0.250 (g=11)

Supplementary Table 7

**Multivariable logistic regression model for hospitalization in symptomatic subgroup (sample size=749)**

|  | **Estimated OR** | **OR 95% CI** | **P value** |
| --- | --- | --- | --- |
| HCQ, yes/no | 0.743 | (0.39,1.37) | 0.355 |
| Age | 1.038 | (1.03,1.05) | <0.001 |
| Gender, male/female | 1.595 | (1.13,2.26) | 0.008 |
| Diabetes, yes/no | 1.562 | (0.99,2.48) | 0.057 |
| Hypertension yes/no | 0.756 | (0.50,1.13) | 0.178 |
| COPD/Asthma, yes/no | 0.951 | (0.59,1.53) | 0.836 |
| Cancer, yes/no | 1.151 | (0.63,2.09) | 0.643 |
| **Disease severity,**  **n (%)** |  |  |  |
| qSOFA Score, 1/0 | 0.184 | (0.13,0.26) | <0.001 |

Note : Test of model goodness of fit shows a good fit with p-value=0.339 (g=10)

Supplementary Figure 2

**Hospitalization according to Hydroxychloroquine Exposure Among Cohort with Fever, Cough or Shortness of Breath at Time of Evaluation**

**Cumulative prevalence of hospitalization among mildly symptomatic COVID-19 patients who self-report at least fever, cough or shortness of breath at time of evaluation according to outpatient exposure to hydroxychloroquine. HCQ=hydroxychloroquine.**

Supplementary Table 8

A) Matched multivariable logistic regression model for hospitalization in age subgroup (sample size=282)

|  | **Estimated OR** | **OR 95% CI** | **P value** |
| --- | --- | --- | --- |
| HCQ, yes/no | 0.489 | (0.17,1.32) | 0.171 |
| Gender, male/female | 0.919 | (0.50,1.69) | 0.786 |
| Diabetes, yes/no | 1.153 | (0.58,2.30) | 0.686 |
| Hypertension yes/no | 0.744 | (0.37,1.48) | 0.4 |
| COPD/Asthma, yes/no | 1.016 | (0.45,2.25) | 0.968 |
| Cancer, yes/no | 0.798 | (0.36,1.76) | 0.578 |
| **Presenting Symptoms, n (%)** |  |  |  |
| Fever, yes/no | 1.17 | (0.57,2.37) | 0.663 |
| Cough, yes/no | 1.003 | (0.47,2.08) | 0.994 |
| Shortness of Breath, yes/no | 11.298 | (5.68,23.84) | <0.001 |
| **Disease severity, n (%)** |  |  |  |
| SOFA Score, 1/0 | 0.187 | (0.10,0.35) | <0.001 |

Note : Test of model goodness of fit shows a good fit with p-value=0.622 (g=12)

B) Matched multivariate logistic regression model with the stepwise (AIC based) variable selection procedure for hospitalization in age subgroup(sample size=282)

|  | **Estimated OR** | **OR 95% CI** | **P value** |
| --- | --- | --- | --- |
| HCQ, yes/no | 0.424 | (0.15,1.10) | 0.087 |
| Diabetes, yes/no | 1.122 | (0.58,2.18) | 0.734 |
| **Presenting Symptoms, n (%)** |  |  |  |
| Shortness of Breath, yes/no | 11.544 | (6.26,22.27) | <0.001 |
| **Disease severity, n (%)** |  |  |  |
| SOFA Score, 1/0 | 0.192 | (0.10,0.35) | <0.001 |

Note : Test of model goodness of fit shows a good fit with p-value=0.865 (g=6)

C) Matched regression model with variables selected by Lasso in age subgroup (sample size=282)

|  | **Estimated OR** | **OR 95% CI** | **P value** |
| --- | --- | --- | --- |
| HCQ, yes/no | 0.423 | (0.15,1.11) | 0.088 |
| **Presenting Symptoms, n (%)** |  |  |  |
| Shortness of Breath, yes/no | 10.267 | (5.67,19.27) | <0.001 |
| **Disease severity, n (%)** |  |  |  |
| SOFA Score, 1/0 | 0.198 | (0.11,0.36) | <0.001 |

Note : Test of model goodness of fit shows a good fit with p-value=0.700 (g=5)

Supplementary Table 9

Univariate logistic regression in HCQ subgroup with symptoms (sample size=69)

|  | **Estimated OR** | **OR 95% CI** | **P value** |
| --- | --- | --- | --- |
| Days From symptom onset to TRT, >2/<=2 | 3.429 | (0.57,66.01) | 0.262 |
